# MRI to Discriminate Pediatric MS from ADEM

**DOI:** 10.15844/pedneurbriefs-29-2-4

**Published:** 2015-02

**Authors:** Jennifer P. Rubin

**Affiliations:** 1 Division of Neurology, Ann & Robert H. Lurie Children's Hospital of Chicago, Chicago, IL; 2 Departments of Pediatrics and Neurology, Northwestern University Feinberg School of Medicine, Chicago, IL

**Keywords:** Multiple Sclerosis, Acute Disseminated Encephalomyelitis, ADEM, Susceptibility-Weighted Imaging

## Abstract

Investigators from Washington University School of Medicine and University of Florida College of Medicine, report that susceptibility-weight imaging (SWI) may be useful in differentiating initial presentation of pediatric multiple sclerosis (MS) from acute disseminated encephalomyelitis (ADEM).

Investigators from Washington University School of Medicine and University of Florida College of Medicine, report that susceptibility-weight imaging (SWI) may be useful in differentiating initial presentation of pediatric multiple sclerosis (MS) from acute disseminated encephalomyelitis (ADEM). MRI brain performed on a 3-T scanner including SWI obtained within 6 months of diagnosis in 18 patients, 8 with ADEM (mean age 5.6 years) and 10 with MS (mean age 15.4), were included for analysis. FLAIR images were coregistered with the SWI images, and each brain lesion was analyzed for its SWI characteristics. The patients ultimately diagnosed with MS had a significantly higher percentage of FLAIR lesions which also demonstrated SWI abnormalities. The MS patients had a larger number of FLAIR hyperintense lesions than the ADEM patients, but this was not statistically significant (*P* = 0.07). The median percentage of lesions identified on SWI with MS diagnosis was 0.22, and for those with ADEM diagnosis was 0 (*P* = 0.04). An optimal discriminator was 0.2 to separate the two groups based on the SWI findings; those with a percentage of lesions visible on SWI <0.2 were likely to have ADEM, and those >0.2 were likely to have multiple sclerosis. [1]

COMMENTARY. Although the precise prevalence is unknown, an estimated 2 to 5% of all persons with multiple sclerosis (MS) have onset of symptoms before 16 years of age [2]. The major challenge to diagnosing MS in the pediatric population is to distinguish transient demyelinating events from life-long MS, and to differentiate MS from other inflammatory or infectious conditions. Acute disseminated encephalomyelitis (ADEM) is an inflammatory disorder of the CNS presenting with multifocal, mainly white matter, abnormalities of the brain and spinal cord. ADEM occurs more commonly in children than adults, is typically monophasic, and includes encephalopathy. Although ADEM and MS in pediatric patients typically have characteristic clinical presentations, there is considerable overlap. Differentiating ADEM from MS is useful to better predict long term prognosis and risk for recurrent demyelination. There are currently no clinical, MRI or CSF findings that can definitively distinguish ADEM from ADEM-like initial CNS demyelinating events. The 2010 McDonald Criteria for diagnosing multiple sclerosis are not as sensitive or specific in pediatric patients when including those with ADEM [3].

In adults with MS, susceptibility-weighted imaging (SWI) may recognize the presence of iron in MS lesions, visualize lesions missed by conventional imaging techniques, and identify particular lesion characteristics [4]. ADEM patients demonstrated less SWI abnormalities, suggesting there might be less iron deposition in the demyelinating lesions of ADEM.

The recently revised diagnostic criteria for pediatric MS allow the initial demyelinating event to be ADEM, followed by a nonencephalopathic clinical event at least three months after symptom onset, if associated with new MRI lesions fulfilling 2010 Revised McDonald criteria [5]. This current study raises the possibility of incorporating additional neuroimaging, particularly in this patient subset, to differentiate children with ADEM versus those with an initial presentation of MS.
